# Predictors of complications following alloplastic cranioplasty in trauma patients: A multi-center retrospective study

**DOI:** 10.1371/journal.pone.0321870

**Published:** 2025-04-23

**Authors:** Jingguo Yang, Junjie Wang, Chao You, Lu Ma, Junwen Guan

**Affiliations:** Department of Neurosurgery, West China Hospital, Sichuan University, Chengdu, Sichuan province, PR China; University of South Carolina, UNITED STATES OF AMERICA

## Abstract

**Object:**

Although technically simple, cranioplasty following decompressive craniectomy is associated with high complication rates. Assessing the factors that contribute to these complications is essential. The study aimed to present the complications following alloplastic cranioplasty in trauma patients and evaluate the factors that predispose patients to an increased risk of complications.

**Methods:**

The author retrospectively reviewed cranioplasty cases at three institutions following craniectomy for trauma between 01/01/2018 and 31/12/2021. The risk factors included age, sex, smoking history, defect site, defect size, type of alloplastic materials, hydrocephalus after decompressive craniectomy (DC), hydrocephalus shunts before cranioplasty (CP), and the time interval between initial craniectomy and cranioplasty. The cranioplasty complications assessed were postoperative new-onset seizures, reoperation for hematoma, implant failure, and postoperative subgaleal effusion. Multivariate logistic regression analysis was performed to assess these risk factors.

**Results:**

A total of 191 cranioplasties were included in this study, with a major complication rate of 26.2% (50 of 191). In multivariate analysis, the risk factors for major complications were smoking history, titanium cranioplasty, and a time interval to cranioplasty exceeding 3 months. Predictors of new-onset seizures in multivariate analysis included younger age, smoking history (OR = 4.69, *p* < 0.001), titanium cranioplasty (OR = 4.85, *p* = 0.049), and intermediate CP (OR = 5.46, *p* = 0.042). The rates of implant failure and postoperative hematoma were higher when cranioplasty was performed over 3 months or involved titanium cranioplasty. The rate of minor complication, specifically subgaleal effusion, was 18.9% (36 cases), with male sex being a significant variable in multivariate analysis.

**Conclusions:**

This study presented complications and predictors of complications for cranioplasty in trauma patients, which could be incorporated with surgical decision-making for neurosurgeons.

## Introduction

Decompressive craniectomy (DC) is a potentially life-saving surgical intervention used to manage refractory intracranial hypertension in patients suffering from severe traumatic brain injury (TBI). The procedure involves the removal of a portion of the skull to alleviate pressure on the brain, thereby preventing further neurological damage and improving patient survival rates [1–3]. While DC is life-saving, it necessitates a subsequent cranioplasty to restore cranial integrity and protect the brain. Cranioplasty, the surgical repair of the skull defect with autologous or alloplastic materials, aims to not only restore the physical barrier but also improve neurological functions and cosmetic outcomes [4–6]. Despite its benefits, cranioplasty is associated with a high incidence of complications, ranging from 15% to 35% as reported in various studies. These complications include infections, seizures, hematomas, and implant failures, which can significantly impact patient recovery and quality of life [7–16,17,18–20,21]. Consequently, understanding the predictors of these complications is vital for optimizing surgical outcomes and patient care. Researchers have extensively studied factors such as the type of implanted materials and the timing of the cranioplasty, both of which are believed to influence the risk of postoperative complications [7,13–16,22,23,20,24–27,28,29].

Previous studies on cranioplasty complications often included heterogeneous populations, encompassing patients with TBI, ischemic and hemorrhagic strokes, and other conditions. This diversity could bias the reported complication rates due to differing underlying pathologies and patient characteristics. Additionally, many studies suffered from limited sample sizes, reducing the robustness and generalizability of their findings [8–11]. Thus, there is a need for focused research on trauma patients undergoing cranioplasty to provide clearer insights into the factors affecting surgical outcomes.

The type of material used in cranioplasty has been a major focus of investigation [13,14,16,28,29]. Alloplastic materials, such as titanium and polyetheretherketone (PEEK), are commonly used due to their favorable properties and ease of customization. However, each material comes with its own set of potential complications. For instance, titanium mesh, while strong and durable, has been associated with higher rates of implant exposure over time and non-physiological heat conduction. PEEK, on the other hand, is known for its biocompatibility and lower complication rates, but its long-term performance requires further study. The timing of cranioplasty after DC is another contentious issue. Early cranioplasty, generally performed within three months after the initial surgery, has been reported to enhance neurological outcomes. However, literature also indicates that this timing is linked with higher complication rates, including infections and postoperative hematoma [22,20,24,25]. Balancing these risks to determine the optimal timing for cranioplasty remains a critical challenge for neurosurgeons.

In light of these considerations, this study aims to elucidate the predictors of complications following alloplastic cranioplasty specifically in trauma patients. By focusing on a homogeneous cohort and examining factors such as the type of implant material and the timing of the procedure, we seek to identify conditions that minimize postoperative complications and improve patient outcomes. This research not only contributes to the existing body of knowledge but also provides practical insights for clinical decision-making in neurosurgical practice.

## Methods

This study is a multi-center, retrospective cohort study that was conducted to elucidate the predictors of complications following alloplastic cranioplasty specifically in trauma patients. The data used in this study were accessed by JY and JW between 01/01/2022 and 01/01/2023. Patients were identified through electronic medical records, and follow-up data were collected via telephone interviews, outpatient visits, or letters to ascertain outcome conditions. Data were collected from three hospitals over a period from 01/01/2018–31/12/2021. The accuracy of the data was ensured by having two authors independently review the medical records.

This retrospective study involving human participants has been reviewed and approved by the Biomedical Research Ethics Committee of West China Hospital, Sichuan University (NO. 2021–1472. Date: October 2021).

The inclusion criteria for the cohort study were severe TBI requiring a DC due to trauma, a craniectomy > 6 cm in diameter, and consent to participate in this study. Electronic medical records (clinical, radiological, follow-up, and outcome data) had to be available. Patients were excluded if they underwent craniectomy for other causes, lost follow-up, had less than 6 months of follow-up, or underwent suboccipital cranioplasty.

Data abstracted from electronic medical records included sex, age at cranioplasty, risk factors (e.g., active smoking), injury cause (road traffic incident, accidental falls, blunt trauma, others), defect site (classified as unilateral or bilateral), size of the defect, time interval between initial craniectomy and cranioplasty, hydrocephalus after DC, hydrocephalus shunts before CP, type of cranioplasty materials used (classified as titanium or PEEK), and follow-up time. The size of the defect was calculated by a formula, π×long axis×short axis. As for hydrocephalus, we only recorded those with hydrocephalus before the CP operation. The diagnosis of hydrocephalus was based on clinical symptoms and characteristic findings on CT imaging. For a history of posttraumatic hydrocephalus shunts surgery, we define it as hydrocephalus shunts before CP. The interval between DC and CP was classified into three categories based on a recent international consensus: early CP (< 3 months), intermediate CP (3–6 months), and delayed CP (≥ 6 months) for research purposes [30].

Postoperative complications related to cranioplasty were categorized as major or minor. Major complications were defined as those necessitating a second operation or long-term medication and included postoperative intracranial hematoma requiring reoperation, postoperative new-onset seizures, and implant failure (due to infection or implant exposure necessitating removal). Minor complications, which could be managed conservatively, included postoperative subgaleal effusion. This is defined as epidural or subdural effusion presenting with clinical signs and symptoms of increased intracranial pressure, with or without midline shift on postoperative CT scan.

All Statistical analysis was performed using SPSS Version 26.0 for Mac (SPSS Inc., Chicago, Ill.). Data are expressed as mean ± standard deviation (SD) and median (IQR) for continuous variables and as frequency (percentage) for categorical variables. Univariate and multivariate logistic regression analysis were used to explore potential factors in the major complications group and each subgroup. To develop a clinical prediction model for major complications, the following variables were included: age, sex, smoking history, defect site, defect size, materials of CP, hydrocephalus after DC, hydrocephalus shunts before CP and timing of CP. The performance of models was assessed by the receiver-operating-characteristic (ROC) curve and the area under the curve (AUC). All statistical tests were two-tailed, with p-values < 0.05 considered statistically significant. Confidence intervals were calculated as 95% CI.

## Results

### Baseline characteristics

A total of 191 patients met the inclusion criteria. The mean age of the patients was 38.6 ± 14.3 years, with 83.8% being male. The median follow-up time from decompressive craniectomy to cranioplasty was 14.6 months. The causes of initial injury were primarily road-traffic incidents (40.8%), accidental falls (30.9%), blunt trauma (7.9%), and others (20.4%). Alloplastic materials used for cranioplasties included titanium (83.2%) and PEEK (16.8%). Preoperative hydrocephalus was present in 14.7% of patients, and 4.7% underwent permanent cerebrospinal fluid diversion before cranioplasty. The majority of cranioplasties were performed on unilateral decompressive craniectomy (92.7%). The timing interval was stratified into three groups for comparison: Early CP (less than 3 months), Intermediate CP (3–6 months), and Delayed cranioplasty (more than 6 months). Thus, 27 patients underwent early cranioplasty, 90 during 3–6 months, and 74 were in the group of delayed CP.

During long-term follow-up, the mortality rate was 1.57%, all resulting from non-surgical factors. Overall, 50 patients (26.2%) experienced at least one major complication, including new-onset seizures (18.9%), postoperative hematoma necessitating operation evacuation (3.1%), and implant failure requiring implant explantation (5.8%). The minor complication of subgaleal effusion was found in 36 patients (18.9%) and could be managed conservatively or with drainage. Among these, 24 patients had subgaleal effusion as the only complication without any major complication. All the demographic information is included in Table 1.

**Table 1:** Clinical characteristics of the patients at baseline.

Variable	All (n = 191)	Major complications* (n = 50)	Minor complications^#^ (without major complications) (n = 24)	No complications (n = 117)
Mean age, mean (SD)	38.6 (14.3)	37.0 (12.8)	37.4 (14.3)	39.5 (14.9)
Sex, N (%)				
Male	160 (83.8)	42 (84)	18 (75)	100 (85.5)
Female	31(16.2)	8 (16)	6 (25)	17 (14.5)
Smoking history, N (%)	66 (34.6)	25 (25)	10 (41.7)	31 (26.5)
Injury cause, N (%)				
1.1 Road-traffic incident	78 (40.8)	26 (52)	10(41.6)	42 (35.9)
1.2 Accidental falls	59 (30.9)	11 (28.0)	8 (33.3)	40 (34.2)
1.3 Blunt trauma	15 (7.9)	3 (6.0)	2(8.3)	10 (8.6)
1.4 Others	39 (20.4)	10 (20.0)	4 (16.7)	25 (21.4)
Defect size, median (IQR)	86.4 (62.8 - 113.0)	84.8 (65.1–113.6)	93.4 (63.4-112.3)	86.4 (62.8-114.2)
Defect site, N (%)				
Bilateral	14 (7.3)	7 (14)	1 (4.17)	5 (4.3)
Unilateral	177 (92.7)	43 (86)	23 (95.8)	112 (95.7)
Hydrocephalus after DC, N (%)	28 (14.7)	8 (16)	7 (29.2)	13 (11.1)
Hydrocephalus shunts before CP, N (%)	9 (4.7)	2 (4)	4 (16.7)	3 (2.6)
Materials, N (%)				
Titanium	159 (83.2)	46 (92)	18 (75)	95 (81.2)
PEEK	32 (16.8)	4 (8)	6 (25)	22 (18.8)
Interval between DC and CP, median (IQR)	5.2 (3.5 - 9.0)	5.8 (4.0- 10.0)	4.7 (3.3 - 10.0)	5.0 (3.4 - 8.2)
Timing of CP (m), N (%)				
Early CP (< 3)	27 (14.1)	2 (4)	6 (25)	19 (16.2)
Intermediate CP (3–6)	90 (47.1)	28 (56)	9 (37.5)	53 (45.3)
Delayed CP (> 6)	74 (38.7)	20 (40)	9 (37.5)	45 (38.5)
follow-up time (m), median (IQR)	14.6 (8.5, 23.5)	18.5 (11.0-26.9)	12.0 (7.5 - 23.9)	14.0 (8.0 - 20.0)

SD, standard deviation; IQR = Interquartile Range; DC, decompressive craniectomy; CP, cranioplasty; PEEK, Polyetheretherketone.

*Major complications include at least one of the following: new-onset seizures, postoperative hematoma, and implant failure. #Minor complication includes subgaleal effusion.

## Factors influencing complications

### Overall complications

In univariate analysis, smoking history (OR 2.49, 95% CI = 1.35–4.60, *p* = < 0.01) was associated with postoperative overall complication. There is a trend indicating a difference in bilateral defect size (OR 3.10, 95% CI = 1.00–9.65, *p* = 0.051). However, only the smoking history was significantly associated with overall complications in multivariate logistic analysis. No other factors were significantly associated with postoperative overall complications in this multivariate logistic analysis. The logistic regression analysis are displayed in S1 Table.

### Major complications

**Fig 1:**
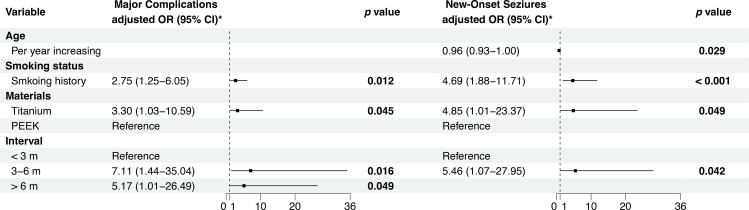
Forest plot presenting the significant results of the multivariate logistic regression analysis for major complications and new-onset seizures. PEEK, Polytheretherketone.

The rate of overall major complications was 26.2% (36 of 191). The proportion of each complication is summarized in Table 2. The most common postoperative major complication was new-onset seizures (26.2% in all patients). In univariate analysis, factors included smoking history, bilateral cranial defect, intermediate and delayed cranioplasty were associated with major complications after CP. In multivariate analysis, smoking history (OR 2.75, 95% CI = 1.25–6.05, *p* = 0.021), intermediate and delayed CP [(OR = 7.11, 95% CI = 1.44–35.04, *p* = 0.016), OR = 5.17, 95% CI = 1.01–26.49, *p* = 0.049)], and titanium mesh CP (OR 3.30, 95% CI = 1.03–10.59, *p* = 0.045) predicted major complications, see Table 3 and Fig 1. We developed a clinical prediction model for major complications, as shown in S1 Figure. It demonstrated good discriminatory ability, distinguishing patients with and without major complications with an AUC of 0.72 (95% confidence interval [CI]: 0.64–0.80).

**Table 2:** Complications following Cranioplasty.

Complication	All, N (%)	Titanium cranioplasty, N (%)	PEEK cranioplasty, N (%)
Major complications#	50 (26.2)	46 (28.9)	4 (12.5)
Seizure	36 (18.9)	34 (21.4)	2 (6.3)
Hematoma	6 (3.1)	5 (3.1)	1 (3.1)
Implant failure	11 (5.8)	10 (6.3)	1 (3.1)
Minor complication			
Subgaleal effusion	36 (18.9)	29 (18.2)	7 (21.9)

PEEK, Polyetheretherketone

**Table 3:** Factors contributing to major complications.

Variable	Univariate analysis			Multivariate analysis		
	OR	95% CI	*P* value	OR	95% CI	*P* value
Major complications*						
Age (per year increasing)	0.99	0.97-1.01	0.350	0.98	0.95-1.01	0.141
Sex						
Male	1.02	0.43-2.46	0.959	0.65	0.24-1.76	0.399
Female	Ref.					
Smoking history	**2.44**	**1.26-4.73**	**< 0.01**	**2.75**	**1.25-6.05**	**0.012**
Defect site						
Unilateral	Ref.					
Bilateral	**3.12**	**1.04-9.39**	**0.043**	2.69	0.82-8.87	0.103
Defect size (mm^2^)						
≥ 90	Ref.					
< 90	1.29	0.68-2.47	0.440	1.26	0.62-2.55	0.517
Materials of CP						
Titanium	2.85	0.95-8.58	0.063	**3.30**	**1.03-10.59**	**0.045**
PEEK	Ref.					
Hydrocephalus after DC	1.15	0.47-2.81	0.755	0.81	0.27-2.45	0.709
Hydrocephalus shunts before CP	0.80	0.16-3.97	0.783	0.86	0.11-6.89	0.889
Timing of CP (m)						
Early CP (< 3)	Ref.					
Intermediate CP (3–6)	**5.65**	**1.25-25.50**	**0.024**	**7.11**	**1.44-35.04**	**0.016**
Delayed CP (> 6)	**4.63**	**1.00-21.36**	**0.049**	**5.17**	**1.01-26.49**	**0.049**
**New-onset Seizures**						
Age (per year increasing)	0.99	0.96-1.01	0.304	**0.96**	**0.93-1.00**	**0.029**
Sex						
Male	2.43	0.69-8.47	0.165	1.19	0.30-4.71	0.810
Female	Ref.					
Smoking history	**3.96**	**1.86-8.44**	**< 0.001**	**4.69**	**1.88-11.71**	**< 0.001**
Defect site						
Unilateral	Ref.					
Bilateral	2.62	0.82-8.35	0.104	2.45	0.66-9.01	0.179
Defect size (mm^2^)						
≥ 90	Ref.					
< 90	1.42	0.68-2.95	0.351	1.27	0.56-2.90	0.567
Materials of CP						
Titanium	4.08	0.93-17.94	0.063	**4.85**	**1.01-23.37**	**0.049**
PEEK	Ref.					
Hydrocephalus after DC	0.93	0.33-2.63	0.885	0.67	0.19-2.38	0.532
Hydrocephalus shunts before CP	0.53	0.06-4.34	0.550	0.41	0.03-5.99	0.511
Timing of CP (m)						
Early CP (< 3)	Ref.					
Intermediate CP (3–6)	4.29	0.94-19.54	0.060	**5.46**	**1.07-27.95**	**0.042**
Delayed CP (> 6)	2.18	0.45-10.56	0.332	2.09	0.38-11.68	0.400

DC, decompressive craniectomy; CP, cranioplasty; PEEK, Polyetheretherketone. *Major Complications include new-onset seizures, postoperative hematoma, implant failure, and subgaleal effusion.

### New-onset Seizures

Patients who had seizures before cranioplasty were excluded from the analysis. Multivariate analysis showed a significantly higher rate of new-onset seizures if cranioplasty was performed at 3–6 months after initial craniectomy (OR = 5.46, CI = 1.07–27.95, *p* = 0.042). Additionally, other significant factors associated with postoperative new-onset seizure were the type of alloplastic material, with a higher rate of seizure when the cranioplasty was performed using titanium mesh (OR = 4.85, 95% CI = 1.01–23.37, *p* = 0.049), smoking history (OR = 4.69, 95% CI = 1.88–11.71, *p* = <0.001). Surprisingly, the increasing age is associated with lower rate of seizures even if this factor was not statistically significant in univariate analysis, see Table 3 and Fig 1.

### Hematoma evacuation and implant failure

Due to the low incidence rates of hematoma (3.14%, 6 cases) and implant failure (5.76%, 11 cases), reliable analyses cannot be conducted using univariate and multivariate analyses. Consequently, we have presented the distribution characteristics of these cases in the S2 Table. and S3 Table.

### Subgaleal effusion

Subgaleal effusion-epidural or subdural effusion with clinical signs and symptoms of increased intracranial pressure with or without midline shift on postoperative CT scan, was a minor complication which could be managed conservatively. Univariate analysis revealed that hydrocephalus shunts before CP (OR = 6.09, 95% CI = 1.55–23.97, *p* = 0.010) was associated with a higher risk, while hydrocephalus after DC showed a trending difference (OR = 2.39, 95% CI = 0.98–5.83, *p* = 0.057). Other factors did not reach statistical significance. In multivariate analysis, only female gender predicted a higher rate of postoperative subgaleal effusion, see Table 4.

**Table 4:** Factors contributing to subgaleal effusion (minor complications).

Variable	Univariate analysis			Multivariate analysis		
	OR	95% CI	*P* value	OR	95% CI	*P* value
Subgaleal effusion						
Age (per year increasing)	1.00	0.97-1.02	0.747	0.99	0.96-1.02	0.566
Sex						
Male	0.50	0.21-1.20	0.118	**0.33**	**0.12-0.89**	**0.028**
Female	Ref.					
Smoking history	1.46	0.69-1.46	0.321	1.80	0.75-4.35	0.192
Defect site						
Unilateral	Ref.					
Bilateral	1.81	0.54-6.15	0.340	1.40	0.36-5.45	0.629
Defect size (mm^2^)						
≥ 90	Ref.					
< 90	0.71	0.34-1.48	0.362	0.82	0.38-1.78	0.610
Materials of CP						
Titanium	0.80	0.31-2.02	0.632	0.69	0.24-1.97	0.487
PEEK	Ref.					
Hydrocephalus after DC	**2.39**	**0.98-5.83**	**0.057**	1.69	0.48-5.92	0.412
Hydrocephalus shunts before CP	**6.09**	**1.55-23.97**	**0.010**	3.12	0.49-19.78	0.227
Timing of CP (m)						
Early CP (< 3)	Ref.					
Intermediate CP (3–6)	0.48	0.18-1.29	0.142	0.39	0.13-1.18	0.095
Delayed CP (> 6)	0.51	0.18-1.40	0.191	0.37	0.11-1.21	0.100

DC, decompressive craniectomy; CP, cranioplasty; PEEK, Polyetheretherketone.

## Discussion

As almost all patients surviving decompressive craniectomy require a second cranioplasty, it has become a common procedure in neurosurgery. Although technically simple, cranioplasty is associated with high complication rates ranging from 15% to 35% [7–16,17,18,19,31,21]. Therefore, it is reasonable to identify, understand, treat and prevent the complications when they arise. The cause of craniectomy was subarachnoid hemorrhage (SAH), stroke, TBI and others, and the heterogeneity of the underlying pathologies might bias the complication rates after cranioplasty [11]. Consequently, we excluded patients who underwent craniectomy for other causes. The complications in our study included major and minor ones, with the former consisting of new-onset seizures, implant failure, and hematoma. Additionally, we included subgaleal effusion as a minor complication, which was managed conservatively or with drainage. This complication has been less reported in previous studies, with some researchers considering it a delayed allergic reaction after PEEK cranioplasty [17]. However, we did not include hydrocephalus as a post-cranioplasty complication because post-traumatic hydrocephalus has been reported as a common complication after decompressive craniectomy [32]. We considered it preexisting hydrocephalus not clinically apparent after the cranioplasty surgery. In this study, 28 patients (14.7%) developed hydrocephalus after decompressive craniectomy; nine patients underwent shunt surgery, and 19 patients had shunt placement after cranioplasty. The overall major complications rate in our study was 26.2%, which is similar to that reported in previous literature. The rate of subgaleal effusion was 18.9%. The purpose of this study was to identify factors contributing to complications, focusing on the time interval and type of alloplastic materials in trauma settings.

Surgery-specific and patient-specific factors are reported to be important predictors of cranioplasty complications [7–10,15,16,19]. The search for ideal implanted materials has continued throughout the development of cranioplasty, with some reports indicating that the type of implant material contributes to complications following cranioplasty [13–15]. Moreover, the interval between craniectomy and cranioplasty has always been a subject of debate, with some studies finding the timing of cranioplasty to be an independent risk factor for complications [7–9,11,12,23,18]. Subgaleal effusion is a less studied complication, and its predictors have not been previously examined. In our study, in addition to demographic data, we included smoking status, defect site, defect size, hydrocephalus after DC, hydrocephalus shunts before CP, timing to cranioplasty and type of cranioplasty materials.

### Risk factors for Overall complications, Major complications, and related factors

Although some studies have shown that age [10,19] is associated with a higher risk of complications, Our study found that younger age is a risk factor for new-onset seizures. This may be due to the retrospective nature of our study, which introduces a certain degree of selection bias, combined with an insufficient sample size, both of which may contribute to these results. Additionally, due to the limited sample size, this study only included key factors in the regression model analysis and did not account for confounding factors such as the presence of underlying comorbidities in patients. These unaccounted factors might also influence the analysis results.

Smoking has been reported as a risk factor in previous study [8,19]. In our study, both univariate and multivariate analysis revealed that smoking was related to major complications and new-onset seizures. Likewise, according to our results, smoking is the only independent risk factor for overall complications (S1 Table). Smoking adversely impacts wound healing through various mechanisms. Nicotine triggers vasoconstriction, which reduces blood flow and oxygen delivery to tissues—both essential for would healing. Additionally, smoking impairs fibroblast function and collagen synthesis, crucial components of tissue repair. These factors contribute to an increased incidence of surgical site infections and implant failure in smokers. Moreover, smoking is associated with systemic inflammation and oxidative stress, which can heighten neuronal excitability and increase the risk of seizures[33,34]. Overall, the neurosurgeons should recognize these relevant factors and take active measures to prevent the potential complications, such as implementing smoking cessation programs.

To data, the optimal interval between craniectomy and cranioplasty remains unclear. Most studies rely on two-group comparison and categorize cranioplasty timing into “early” and “late”, with 3 or 6 months as the chosen time points. Some recent studies have shown that early cranioplasty could improve neurological function [22,20], whereas others suggest early cranioplasty is related to higher complication rates [24,25]. Meanwhile, other studies reported late cranioplasty is associated with higher complications rates [26], and some showed no difference in complication rate between early and late groups [27,35]. Our study classifies time intervals as early CP (less than 3 months), Intermediate CP (3–6 months), and Delayed cranioplasty (more than 6 months) according to a recent consensus [30]. The results indicate that cranioplasty performed more than 3 months after the initial surgery is a risk factor for postoperative major complications and new-onset seizures, consistent with previous reports [7]. The majority of our patients underwent cranioplasty more than 3 months after the initial surgery, and none of the patients who had cranioplasty within 3 months developed implant failure. This finding aligns with previous reports [36], while work of some researchers shows opposite trend [37]. In the past, some recommended waiting at least one year or waiting as long as possible to allow sufficient recovery for patients [36]. However, our study found that late cranioplasty was clearly associated with a high rate of complications, indicating that early cranioplasty is beneficial in reducing the occurrence of major complications. More studies are needed to determine the effect of timing on neurological function outcome [38].

Cranioplasty has undergone many advancements in the search for ideal materials to improve outcomes. Although autologous bone was always regarded as gold standard in skull reconstruction, bone flap resorption is a common complication [28,39]. Some recent studies have reported that autologous bone grafts appear to carry an increased reoperation rate [16,29,40]. Zaed et al. conducted a systematic review focusing on the pediatric population, comparing autologous and heterologous outcome. Their findings revealed that heterologous materials are superior to autologous bone for CR in children [41]. Also, Signorelli et al. conducted a comprehensive analysis of factors leading to bone flap resorption after decompressive craniectomy. Their study concluded that bone flap fragmentation, TBI etiology, and younger age increase the risk of bone flap resorption. This raises the question of whether alloplastic materials should be recommended for these patient population [42]. Various methods of alloplastic cranioplasty have been developed, each with different characteristics [13,14,43]. In this study, titanium mesh and PEEK were the implanted materials. Titanium mesh showed a higher incidence of overall major complications, particularly seizures. A possible explanation for this is titanium’s high thermal conductivity, which allows environment temperature fluctuations to be transmitted to the brain. These may influence neuronal homeostasis, thereby increasing risk of seizures. Also, we found the incidence of implant failure and hematoma were higher in titanium CP [(90.9% vs. 9.1%), (83.3% vs. 16.7%), respectively], as shown in S3 Table. However, considering the limited sample in the two groups, no logistic regression analysis was done. Our previous study also demonstrated that cranioplasty with PEEK had advantages in brain function improvement and fewer complications [13]. Therefore, we consider PEEK cranioplasty to be more ideal for cranial reconstruction. But is PEEK necessarily the best choice? Although PEEK CP has various advantages, our previous studies have also shown that PEEK CP requires longer surgical time and costs nearly four times as much as titanium. Therefore, it is necessary to consider the cost-effectiveness and the patients’ financial situation. More research data are needed in the future [44, 45].

Some studies have found bilateral defect site to be associated with cranioplasty complications, such as seizure and death [31,46]. In our study, we also found that patients with bilateral cranioplasty had a higher rate of major complications. However, the statistical difference was not present in the multivariate analysis. We hypothesize multiple operations, longer incisions, thin soft tissue, and involvement of the skull base contribute to these complications, as observed in previous studies [47,48].

Seizures that follow cranioplasty was the most common complication in our study, with incidence of 18.9% (36 cases). This may be due to the fact that the included patients underwent decompressive craniectomy for TBI, as some studies have shown that TBI before cranioplasty increased the risk of seizures [8,21]. Additionally, the large majority of patients had cranioplasty performed more than 3 months after the initial surgery, which may also be another reason, as a longer interval between decompressive and cranioplasty increases the likelihood of postoperative seizures [7]. Other contributing factors might include the use of bipolar coagulator during the operation and scar-related epilepsy.

### Risk factors for subgaleal effusion and related factors

Subgaleal effusion, while common, has received less attention in similar publications. Some authors speculated it as delayed allergic reactions particularly in PEEK cranioplasty [17]. We found no significant difference in the incidence of subgaleal effusion across different materials (Table 2). Our study suggests that there are associations between hydrocephalus shunts before CP, and postoperative subgaleal effusion; however, this difference was only observed in the univariate analysis and did not reach statistical significance in the multivariate analysis. Yao [49] also reported that a history of ventriculoperitoneal shunt was an independent risk factor for overall complications including subgaleal effusion. The disturbance of cerebrospinal fluid circulation could be the possible reason. Planning cranioplasty in conjunction with hydrocephalus remains a significant challenge. Our findings align with a recent consensus emphasizing the need for standardized diagnostic and surgical protocols for both cranioplasty and post-traumatic hydrocephalus. The recommended management sequence prioritizes cranioplasty as the primary intervention [50]. Additionally, cranioplasty performed within 3 months was identified as a risk factor. Therefore, the adjustment of drainage speed could be important. We also identified sex as a significant variable and found that females had a higher risk of subgeleal effusion, which is consistent with findings of Chang [10]. However, laboratory examinations of the effusion were not performed, and more data are needed in the future to elucidate the mechanisms and outcomes of this complication.

### Limitations and strengths of this study

This study has several limitations. First, as a retrospective study, it is subject to possible selection bias. Subsequent analyses will employ Propensity score matching (PSM), which has been widely used to reduce confounding biases in observational studies. Additionally, the study did not include enough patient-specific and surgery-specific risk factors, such as diabetes, hypertension, and dura tearing during the cranioplasty surgery, and it lacked stratification of some factors. Furthermore, most patients underwent cranioplasty more than three months after the initial surgery, and the small sample for “early” cranioplasty was relatively small. Moreover, the classification of subgaleal effusion as a minor complication may not fully reflect its potential severity or its progression to major complications, such as seizures. This categorization could lead to an underestimation of its clinical impact. Future research should focus on stratifying subgaleal effusion based on severity and clinical management requirements to provide a more comprehensive understanding of its impact.

One of the major strengths is its multi-center study design, which allows for a larger and more diverse patient population, thereby improving the robustness of the results. Additionally, by including only patients with TBI, we reduced the heterogeneity of the pathologies of decompressive craniectomy. The large sample size and long-term follow-up allow for robust statistical analysis. Moreover, we analyzed the timing and various alloplastic materials used in cranioplasty, which are two contentious points in cranioplasty. Although this study did not focus on the relationship between the timing of cranioplasty and neurological functions, the effect of timing on neurological outcomes is currently an important area of research. Further prospective studies are required to explore those findings.

## Conclusions

In conclusion, we presented the occurrence of complications following alloplastic material in trauma patients and presented related risk factors. Smoking history was the only independent risk factor for overall complications. Furthermore, we categorized the complications into major complications and minor complications. and complications following alloplastic material in trauma patients included both major and minor complications. Major complications include seizures, hematoma, and implant failure while minor complications refer to subgaleal effusion. The independent predictors of major complications were smoking history, titanium CP, and cranioplasty performed more than 3 months after the initial surgery. The titanium cranioplasty, smoking history and an intermediate CP were identified as risk factors for postoperative new-onset seizures. Additionally, there was association between female sex with subgaleal effusion. While the best answers to these questions should be addressed by prospective studies, the findings of this study provide a basis for surgical decision-make and future investigations regarding cranioplasty.

## Supporting information

S1 Table Factors Contributing to Overall Complication. (DOCX)

S2 Table Characteristics of patients with or without hematoma. (DOCX)

S3 Table Characteristics of patients with or without implant failure. (DOCX)

S1 Figure Receiver operating characteristic curves for the clinical prediction model of major complications. The area under the curve was 0.73 (95% CI:0.65–0.81). The red line represents a test of no discrimination. (PNG)

S1 File Research Proposal. (PDF)
