# Supplementary material for: Predictors of complications following alloplastic cranioplasty in trauma patients: A multi-center retrospective study
Source: PLoS One. 2025 Apr 23;20(4):e0321870. doi: 10.1371/journal.pone.0321870 (PMC12017519; doi:10.1371/journal.pone.0321870)
Supplement: S1 Table — (DOCX) [file pone.0321870.s001.docx]

S1 Table. Factors Contributing to Overall Complication

| Variable | Univariate analysis | | | Multivariate analysis | | |
| --- | --- | --- | --- | --- | --- | --- |
|  | OR | 95% CI | *P* value | OR | 95% CI | *P* value |
| Overall Complications* |  |  |  |  |  |  |
| Age (per year increasing) | 0.99 | 0.97-1.01 | 0.257 | 0.98 | 0.96-1.01 | 0.118 |
| Sex |  |  |  |  |  |  |
| Male | 0.73 | 0.34-1.58 | 0.424 | 0.44 | 0.19-1.05 | 0.066 |
| Female | Ref. |  |  |  |  |  |
| Smoking history | **2.49** | **1.35-4.60** | **< 0.01** | **2.89** | **1.43-5.83** | **0.003** |
| Defect site |  |  |  |  |  |  |
| Unilateral | Ref. |  |  |  |  |  |
| Bilateral | **3.10** | **1.00-9.65** | **0.051** | 2.23 | 0.67-7.43 | 0.193 |
| Defect size (mm^2^) |  |  |  |  |  |  |
| ≥ 90 | Ref. |  |  |  |  |  |
| < 90 | 1.00 | 0.56-1.80 | 0.993 | 1.06 | 0.57-2.00 | 0.848 |
| Materials of CP |  |  |  |  |  |  |
| Titanium | 1.48 | 0.66-3.34 | 0.342 | 1.56 | 0.63-3.84 | 0.336 |
| PEEK | Ref. |  |  |  |  |  |
| Hydrocephalus after DC | 2.03 | 0.91-4.57 | 0.085 | 1.22 | 0.44-3.40 | 0.702 |
| Hydrocephalus shunts before CP | 3.35 | 0.81-13.85 | 0.094 | 2.60 | 0.43-15.81 | 0.299 |
| Timing of CP (m) |  |  |  |  |  |  |
| Early CP (< 3) | Ref. |  |  |  |  |  |
| Intermediate CP (3-6) | 1.66 | 0.66-4.19 | 0.285 | 1.69 | 0.60-4.74 | 0.319 |
| Delayed CP (> 6) | 1.53 | 0.59-3.95 | 0.379 | 1.39 | 0.47-4.10 | 0.556 |

DC, decompressive craniectomy; CP, cranioplasty; PEEK, Polyetheretherketone. *Overall Complications include at least one of the following: new-onset seizures, postoperative hematoma, implant failure and subgaleal effusion.

S2 Table. Characteristics of patients with or without hematoma

| Variable | Hematoma  (n=6) | No hematoma  (n=185) |
| --- | --- | --- |
| Mean age, mean (SD) | 44.4 (15.3) | 38.4 (14.2) |
| Sex, N (%) |  |  |
| Male | 3 (50.0) | 157 (84.9) |
| Female | 3 (50.0) | 28 (15.1) |
| Smoking, N (%) | 2 (33.3) | 64 (34.6) |
| Defect size (mm^2^) |  |  |
| ≥ 90 | 3 (50.0) | 90 (48.6) |
| < 90 | 3 (50.0) | 95 (51.4) |
| Defect site, N (%) |  |  |
| Unilateral | 1 (16.7) | 13 (7.0) |
| Bilateral | 5 (83.3) | 172 (93.0) |
| Hydrocephalus after DC, N (%) | 1 (16.7) | 27 (14.6) |
| Hydrocephalus shunts before CP, N (%) | 0 | 9 (4.9) |
| Materials of CP, N (%) |  |  |
| Titanium | 5 (83.3) | 154 (83.2) |
| PEEK | 1 (16.7) | 31 (16.8) |
| Timing of CP (m), N (%) |  |  |
| Early CP (< 3) | 0 (0) | 27 (14.6) |
| Intermediate CP (3-6) | 3 (50.0) | 87 (47.0) |
| Delayed CP (> 6) | 3 (50.0) | 71 (38.4) |

SD, standard deviation; DC, decompressive craniectomy; CP, cranioplasty; PEEK, Polyetheretherketone.

S3 Table. Characteristics of patients with or without implant failure

| Variable | Implant failure  (n=11) | No Implant failure  (n=180) |
| --- | --- | --- |
| Mean age, mean (SD) | 37.5 (12.3) | 38.7 (14.4) |
| Sex, N (%) |  |  |
| Male | 8 (72.7) | 152 (84.4) |
| Female | 3 (27.3) | 28 (15.6) |
| Smoking, N (%) | 2 (18.2) | 64 (35.6) |
| Defect size (mm^2^) |  |  |
| ≥ 90 | 6 (54.5) | 87 (48.3) |
| < 90 | 5 (45.5) | 93 (51.7) |
| Defect site, N (%) |  |  |
| Unilateral | 2 (18.2) | 12 (6.7) |
| Bilateral | 9 (81.2) | 168 (93.3) |
| Hydrocephalus after DC, N (%) | 2 (18.2) | 26 (14.4) |
| Hydrocephalus shunts before CP, N (%) | 1 (9.1) | 8 (4.4) |
| Materials of CP, N (%) |  |  |
| Titanium | 10 (90.9) | 149 (82.8) |
| PEEK | 1 (9.1) | 31 (17.2) |
| Timing of CP (m), N (%) |  |  |
| Early CP (< 3) | 0 (0) | 27 (15.0) |
| Intermediate CP (3-6) | 2 (18.2) | 88 (48.9) |
| Delayed CP (> 6) | 9 (81.2) | 65 (36.1) |

SD, standard deviation; DC, decompressive craniectomy; CP, cranioplasty; PEEK, Polyetheretherketone.
