# Supplementary figures and images for: Predictors of complications following alloplastic cranioplasty in trauma patients: A multi-center retrospective study
Source: PLoS One. 2025 Apr 23;20(4):e0321870. doi: 10.1371/journal.pone.0321870 (PMC12017519; doi:10.1371/journal.pone.0321870)

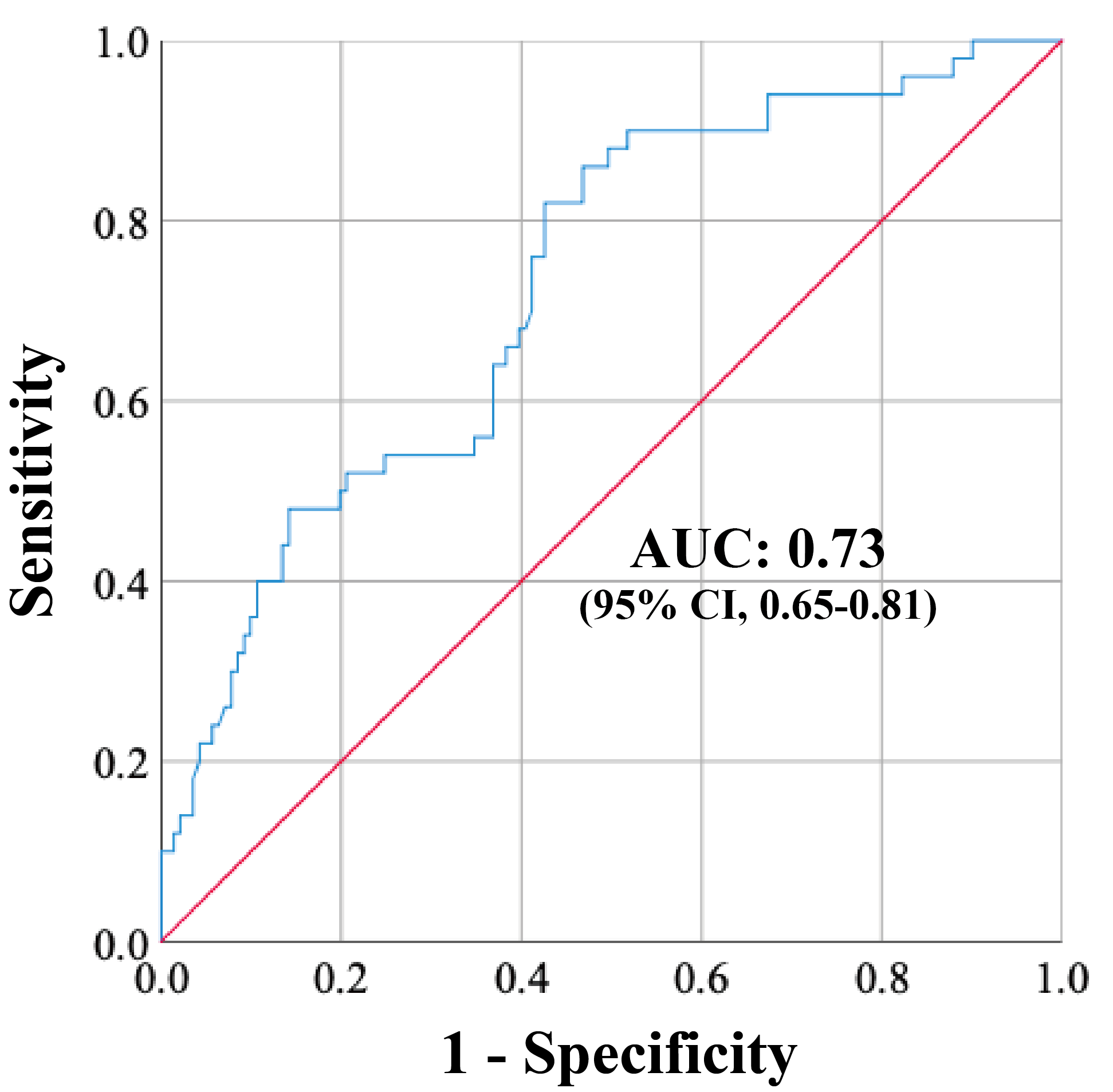

Supplement: S1 Figure — (PNG) [file pone.0321870.s004.png]
