# Supplementary material for: Predictors of complications following alloplastic cranioplasty in trauma patients: A multi-center retrospective study
Source: PLoS One. 2025 Apr 23;20(4):e0321870. doi: 10.1371/journal.pone.0321870 (PMC12017519; doi:10.1371/journal.pone.0321870)
Supplement: S1 File — (PDF) [file pone.0321870.s005.pdf]

Research Proposal

(Non-interventional Clinical Study)

Clinical Research Proposal on the Analysis of Complications after Cranioplasty Following Decompressive Craniectomy

Research Institution: West China Hospital, Sichuan University

Project Leader (Signature):

Responsible Department: Department of Neurosurgery

Contact Number: 18981381916

Research Duration: December 2021 – December 2024

Version Number: V1.0

Version Date: October 26, 2021

Proposal Summary

Study Design

(Multiple choices available)
□ Case-control study
☑ Cohort study
□ Cross-sectional study
□ Randomized controlled trial
□ Blinded method
□ Other:

Type of Study (please check according to the project type)

□ Companion diagnostic reagent kit study
☑ Retrospective study of past clinical data
□ Retrospective study of past clinical specimens
□ Biobank establishment study
□ Cohort establishment study
□ Class I clinical new technology (medical technology with confirmed safety and efficacy, low technical difficulty, and almost no ethical risk)
□ Past case report
□ Non-implantable medical device research (masks, mouth guards, etc.)
□ Other (to be determined by the researcher, please specify:)

Total number of cases

Risk/Benefit Analysis
Not applicable

Risk Assessment
☑ Not greater than minimal risk
□ Greater than minimal risk
Minimal risk: refers to the likelihood and extent of risk expected in the study not exceeding those encountered in daily life or during routine physical examinations or psychological tests.

I. Research Background
Decompressive craniectomy effectively alleviates refractory intracranial pressure caused by brain injury. Secondary cranioplasty is a necessary and common neurosurgical procedure aimed at restoring skull integrity and assisting in the recovery of neurological function. However, despite its relative simplicity, cranioplasty is associated with a high complication rate (15%-35%). Key issues related to cranioplasty include: (1) management of complications following decompressive craniectomy; (2) choice of materials for cranioplasty; (3) interval between decompressive craniectomy and cranioplasty; (4) management of hydrocephalus post-decompressive craniectomy and cranioplasty. Controversies exist, such as the selection of materials for cranioplasty (e.g., autologous bone, titanium, and polyetheretherketone). How does the interval between decompressive craniectomy and cranioplasty affect post-operative complications?

The purpose of this study is to analyze the complications and incidence of cranioplasty following decompressive craniectomy due to brain injury or other causes, identify potential risk factors for these complications, and establish a predictive model to guide clinical treatment.

II. Research Objectives
1. Primary Objectives:
(1) Identify risk factors for complications following cranioplasty.
(2) Focus on potential risk factors for surgical failure post-cranioplasty and construct a related predictive model.

2. Secondary Objectives:
(1) Estimate the incidence of complications post-cranioplasty at West China Hospital.
(2) Explore the optimal timing for cranioplasty.

III. Study Design Type, Principles, and Sample Source
1. Study Design
1.1 Overall Design: This is a single-center, retrospective study that will retrospectively analyze the pre-operative, intra-operative, and post-operative conditions of all patients who underwent cranioplasty following decompressive craniectomy at our department from 2018 to 2021 using the HIS system of our hospital. Long-term follow-up will be conducted to analyze the incidence of various complications post-cranioplasty and identify

corresponding risk factors. Anonymity will be maintained throughout the analysis to protect patient privacy.

1.2 Sample Size: According to literature reports and relevant regression analyses, the sample size will be 10-15 times the number of covariates. Based on previous literature analyzing cranioplasty complications, it is estimated that at least 500 patients meeting the inclusion criteria will be enrolled.

2. Name, Source, Period, Acquisition, Processing, and Disposal of Medical Records/Samples: Data will be obtained through a unified application on the big data platform of our hospital and from the HIS system of West China Hospital and its Shangjin branch, covering patients who underwent cranioplasty following decompressive craniectomy for various reasons from 2010 to 2020. Anonymity will be maintained throughout the analysis to protect patient privacy and data confidentiality.

V. Principles and Requirements of Clinical Research Ethics
This clinical study will adhere to the Helsinki Declaration of the World Medical Association and relevant regulations of the National Health Commission of the People's Republic of China on the ethical review of biomedical research involving humans. The study will only use past case information with personal data removed, posing no risk to participants and not adversely affecting their rights and health. Therefore, informed consent exemption is requested. Research data will be stored at West China Hospital, and researchers, supervisors, and the ethics review committee may review the data. The public report of this research will not disclose participants' personal identities. We will make every effort to protect the privacy and personal information of participants within the legal limits.

VI. Research Progress
- November 2021 – June 2022: Apply for data through the big data platform and complete relevant information via the HIS system. Two researchers will collect and cross-check patient treatment information.
- June 2022 – December 2022: Follow up with patients to understand changes in their current condition.
- January 2023 - January 2024: Organize follow-up results, conduct statistical analysis, build predictive models, and draft research articles.

VII. Participants

Name	Title	Specialty	Task	GCP Training Certificate
Guan Junwen	Chief Physician	Neurosurgery	Experimental Design	✓
Yang Jingguo	None	Neurosurgery	Experimental Design, Data Collection, Statistics, Article Writing	✓
Wang Junjie	None	Neurosurgery	Experimental Data Collection	-

Main References

1. Shepetovsky D et al. Complications of cranioplasty in relationship to traumatic brain injury: a systematic review and meta-analysis. Neurosurg Rev. 2021 Mar 8. doi: 10.1007/s10143-021-01511-7.
2. Zhang Q et al. A Large Multicenter Retrospective Research on Embedded Cranioplasty and Covered Cranioplasty. World Neurosurg. 2018 Apr;112:e645-e651. doi: 10.1016/j.wneu.2018.01.114.
3. Shih FY et al. Risk factors for seizures after cranioplasty. Seizure. 2019;66:15-21. doi: 10.1016/j.seizure.2018.12.016.
4. Morton RP et al. Timing of cranioplasty: a 10.75-year single-center analysis of 754 patients. J Neurosurg.2018 Jun;128(6):1648-1652.
5. 高永祥张晋昕. Logistic 回归分析的样本量确定[J]. 循证医学 201818(2):122-124. DOI:10.12019/j.issn.1671-5144.2018.02.015.
6. Honeybul S Ho KM. Long-term complications of decompressive craniectomy for head injury. J Neurotrauma. 2011 Jun;28(6):929-35. doi: 10.1089/neu.2010.1612.
7. Chaturvedi J et al. Complications of cranioplasty after decompressive craniectomy for traumatic brain injury. Br J Neurosurg. 2016;30(2):264-8.
8. Iaccarino C et al. Consensus statement from the international consensus meeting on post-traumatic cranioplasty. Acta Neurochir (Wien). 2021 Feb;163(2):423-440.
9. Lilja-Cyron A et al. Long-Term Effect of Decompressive Craniectomy on Intracranial Pressure and Possible Implications for Intracranial Fluid Movements. Neurosurgery. 2020 Feb 1;86(2):231-240. doi: 10.1093/neuros/nyz049.
10. Lilja-Cyron A et al. Intracranial pressure before and after cranioplasty: insights into intracranial physiology. J Neurosurg. 2019 Oct 18:1-11.
